# Evaluation of uttroside B, a saponin from *Solanum nigrum* Linn, as a promising chemotherapeutic agent against hepatocellular carcinoma

**DOI:** 10.1038/srep36318

**Published:** 2016-11-03

**Authors:** Lekshmi R. Nath, Jaggaiah N. Gorantla, Arun Kumar T. Thulasidasan, Vinod Vijayakurup, Shabna Shah, Shabna Anwer, Sophia M. Joseph, Jayesh Antony, Kollery Suresh Veena, Sankar Sundaram, Udaya K. Marelli, Ravi S. Lankalapalli, Ruby John Anto

**Affiliations:** 1 Division of Cancer Research, Rajiv Gandhi Centre for Biotechnology, Thiruvananthapuram-695014, Kerala, India; 2 Chemical Sciences and Technology Division, CSIR-National Institute for Interdisciplinary Science and Technology, Thiruvananthapuram-695019, Kerala, India; 3 Department of Pathology, Government Medical College, Thiruvananthapuram-695011, Kerala, India; 4 Division of Organic Chemistry, CSIR-National Chemical Laboratory, Dr. Homi Bhabha Road, Pune-411008, India

## Abstract

We report, for the first time, the remarkable efficacy of uttroside B, a potent saponin from *Solanum nigrum* Linn, against liver cancer. The compound has been isolated and characterized from the leaves of *Solanum nigrum* Linn, a plant widely used in traditional medicine and is a rich resource of several anticancer molecules. Uttroside B, that comprises of β-D-glucopyranosyl unit at C-26 of the furostanol and β-lycotetraosyl unit at C-3, is ten times more cytotoxic to the liver cancer cell line, HepG2 (IC50: 0.5 μM) than sorafenib (IC50: 5.8 μM), the only FDA-approved drug for liver cancer. Moreover, it induces cytotoxicity in all liver cancer cell lines, irrespective of their HBV status, while being non-toxic to normal immortalized hepatocytes. It induces apoptosis in HepG2 cells by down-regulating mainly the activation of MAPK and mTOR pathways. The drastic reduction in HepG2-xenograft tumor size achieved by uttroside B in NOD-SCID mice and substantiation of its biological safety through both acute and chronic toxicity studies in Swiss albino mice warrants clinical validation of the molecule against hepatic cancer, for which, the chemotherapeutic armamentarium currently has limited weapons.

Triterpene and steroid glycosides, commonly referred to as saponins, are isolated primarily from the plant kingdom and exert a wide range of pharmacological properties owing to their large structural diversity^1^. A vast array of saponins have been reported to exhibit antitumor effect against cancer cells originating in different anatomical sites. In natural product research, analysis of the chemotherapeutic efficacy of saponins against various cancer cells is often confined to *in vitro* analysis and structure elucidation^2^. Various plant species of *Solanum* genera have been reported to have considerable amounts of saponins, which exhibit potent anticancer activity against different cancer cell lines^2^,^3^,^4^. *Solanum nigrum* Linn, commonly known as black nightshade, is a medicinal plant member of Solanaceae family, widely used in many traditional systems of medicine^4^. Alcoholic extract of the whole plant has been reported to contain various steroidal saponins, which induce cytotoxicity in different cancer cell lines^5^,^6^,^7^. Two furostanol saponins, uttroside A and B have been reported from the stems and roots of *S. nigrum* Linn^8^. In the present study, we isolated and characterized uttroside B from the leaves of *S. nigrum* Linn and found that the compound exhibits maximum cytotoxicity against liver cancer cells and is ten times more potent than sorafenib, the only FDA-approved drug for liver cancer. Though the cytotoxicity of the compound has been reported in cancer cells of other origins^9^,^10^, this is the first study evaluating its chemotherapeutic efficacy and exploring the molecular mechanisms involved. We have also validated the anticancer potency of the compound *in vivo* using HepG2-xenograft model in NOD-SCID mice and have confirmed its biological safety, both by *in vitro* and *in vivo* studies.

## Results

### The methanolic extract of the leaves of *S. nigrum* Linn contains a bioactive mixture of a saponin and proline (SP), which on further purification yields uttroside B

We conducted a polarity-graded successive extraction of the leaves of *S. nigrum* Linn using hexane, dichloromethane, ethyl acetate and methanol and the cytotoxic effect of the extracts were screened against a panel of human cancer cell lines of different origin by MTT assay. The methanolic extract emerged to be the most cytotoxic and the liver cancer cell line, HepG2 exhibited maximum sensitivity (IC50–37.5 μg/ml) towards the extract followed by the cervical cancer cell line, HeLa (124.2 μg/ml). Later on, the most active methanolic extract was selected for further purification and the most sensitive cell line to the extract, HepG2, was selected for further screening (Supplementary Figure S1A). The methanolic extract (6.3 g) was subjected to fractionation by column chromatography. Among the column fractions subjected to cytotoxicity analysis, ‘fraction f’ turned out to be the most effective (Supplementary Figure S1B).

**Figure 1:**
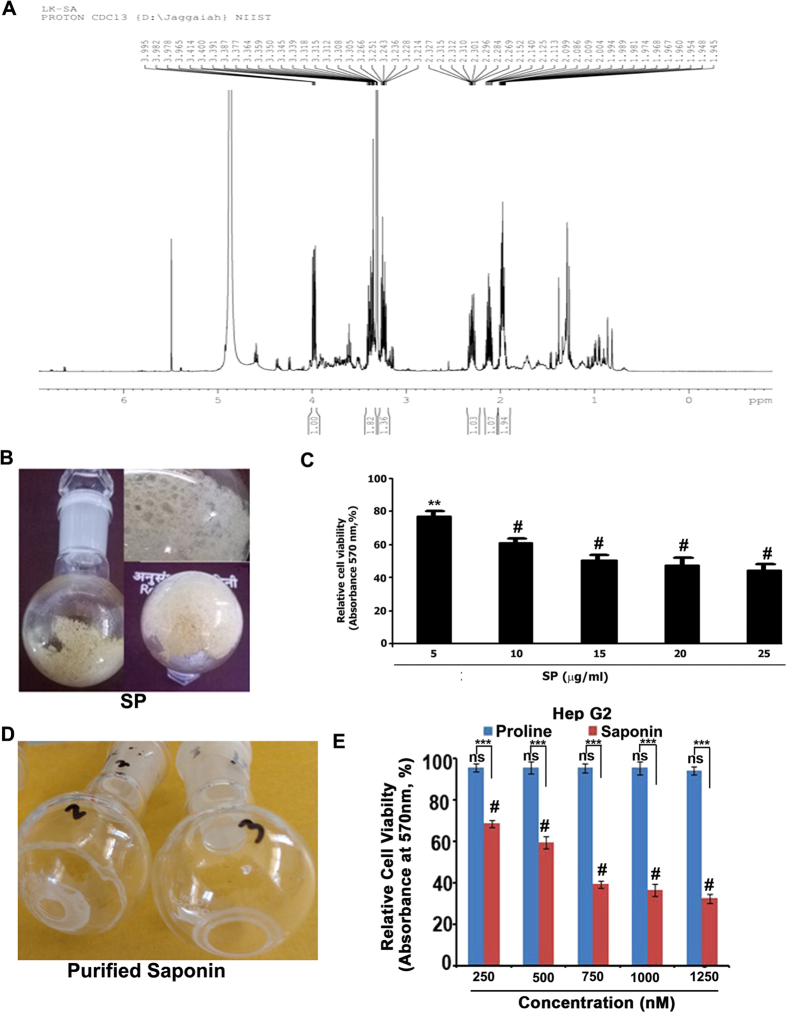
The saponin component is responsible for the cytotoxic effect of the saponin-proline (SP) mixture in HepG2 cells. (**A**) ^1^H-NMR of mixture of proline and saponin (**B**) Pale yellow foamy solid which was a mixture of proline and saponin. This active column mixture isolated from the methanolic extract of *S. nigrum* Linn and this fraction was subjected to vacuum evaporation. (**C**) HepG2 cells were treated with different concentrations of SP and cell viability was assessed by MTT. The error bars represent ±S.D. One-way ANOVA followed by Tukey’s post hoc *t* test analysis was used for statistical comparison between different groups. **P ≤ 0.01; ^#^P ≤ 0.001. (**D**) Saponin, a white solid isolated on vacuum evaporation of the pure active column fraction of methanolic extract of *S. nigrum* Linn (**E**) Comparison of the cytotoxicity of isolated saponin and proline in HepG2 cells. The cells were treated with indicated concentrations of proline and saponin, incubated for 72 h and the cell viability was assessed by MTT assay. Data represent three independent sets of experiments. The error bars represent ±S.D. Two-way ANOVA followed by Tukey’s post hoc *t* test analysis was used for statistical comparison between different groups. ***P ≤ 0.001; ^#^P ≤ 0.001, ns non significant. Symbol *asterisk* (*) represents statistical significance between control and treatment groups where as *hash* (#) represents statistical significance between different treatment groups.

‘Fraction f’, which was identified as a mixture of proline and saponin (SP) by ^1^H-NMR (Fig. 1A) was a pale yellow foamy solid (Fig. 1B) and exhibited a drastic enhancement in cytotoxicity (Fig. 1C; IC50: 10 μg/mL). SP (700 mg) was redissolved in H_2_O (6 mL) and then subjected to purification by reverse-phase preparative HPLC using the gradient program: solvent A (H_2_O) and solvent B (MeOH), linear gradient 0 min 0% B, 5 min 10% B, 10 min 20% B, 15 min 30% B (isolated proline, 130 mg, between 10–15 min), 20 min 50% B, 30 min 60% B, 60 min 80% B, 65 min 90% B, 70 min 100% B. The saponin eluted between 65% to 80% B, monitored over TLC by charring with 15% sulfuric acid in ethanol, was concentrated and lyophilized to afford a white solid (120 mg, Fig. 1D). We compared the cytotoxicity of the isolated proline and saponin in HepG2 cells and found that the saponin is significantly cytotoxic (IC50- 6.08 μg/mL), while proline is not (Fig. 1E), confirming that the saponin is responsible for the cytotoxic effect of SP.

### Characterization of the isolated saponin by Mass and NMR spectroscopy revealed that the bioactive compound is uttroside B, a furostanol glycoside

**Figure 2:**
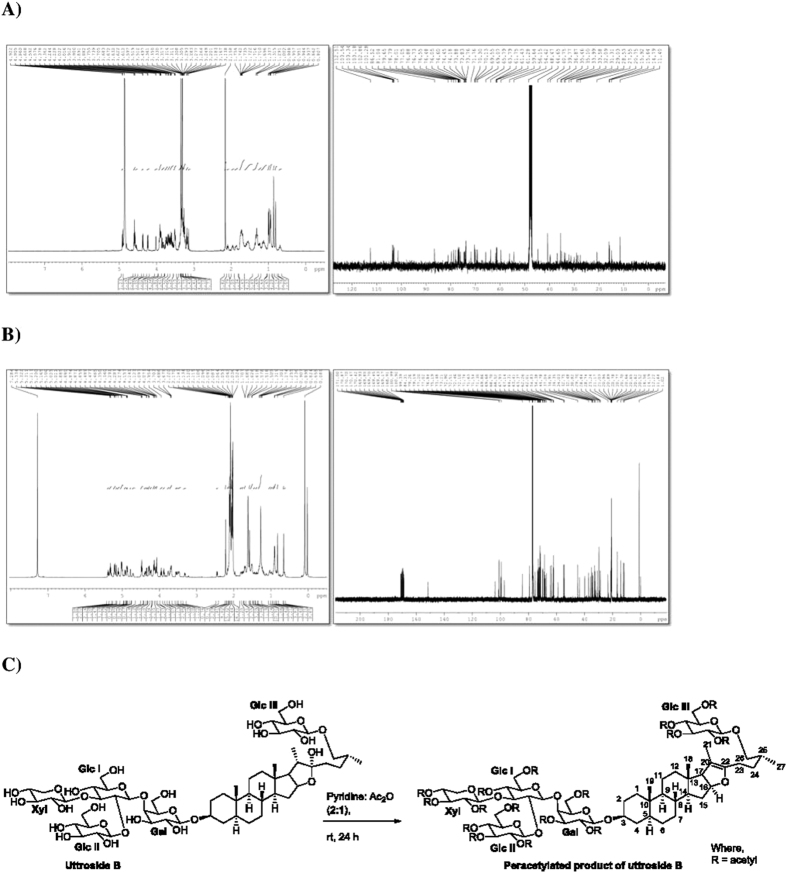
Structure elucidation of uttroside B. (**A**) ^1^H NMR and ^13^C NMR spectra of saponin. (**B**) ^1^H NMR and ^13^C NMR spectra of peracetylated product of saponin. (**C**) Peracetylation of saponin.

Further, we attempted for the structural elucidation of the saponin using ^1^H and ^13^C-NMR experiments, which were initially performed in CD_3_OD (deuterated methanol) solvent. The key information pertaining to steroidal furanose ring include H-21 methyl group at δH 0.99 ppm (3H, d, J = 7 Hz), and hemiketal carbon C-22 at δC 112.5 ppm. Owing to complex pattern of signals arising due to sugars in the region between 3 to 4 ppm, the isolated saponin was peracetylated (Fig. 2A). ^1^H and ^13^C-NMR experiments of peracetylated product of saponin were performed in CDCl_3_ (deuterated chloroform) solvent. Surprisingly, after acetylation the H-21 methyl group exhibited a downfield shift at δH 1.57 ppm (3H, s), and H-17 at δH 2.45 ppm ((Fig. 2B; 1H, d, J = 9.8 Hz, Supplementary Table S1). In ^13^C-NMR, the hemiketal carbon peak disappeared and two additional peaks appeared at δC 103.7 and 151.7 ppm indicating carbons C-20 and C-22, respectively (Fig. 2B). From NMR data, it is clear that a new olefinic bond in the furanose ring formed due to loss of a water molecule during acetylation (SI methods). On the basis of the aforementioned information and available literature, the isolated saponin was identified as uttroside B (Fig. 2C). The HRESIMS data of the isolated saponin analyzed in negative mode showed (M-H)- ion at m/z 1213.6145 indicating a molecular formula C_56_H_93_O_28_. MS-MS analysis in negative mode afforded ions at m/z 1081.5 (M-xyl-H), 919.5 (M-hex-H), 757.4 (M-hex-hex-H), and in positive mode afforded ions at m/z 1235.5 (M-H_2_O + K), 1197.5 (M-H_2_O), 1073.4 (M-H_2_O-hexose + K), 741.4 (M-H_2_O-hex-hex-xyl + H), 579.3 (M-H_2_O-hex-hex-xyl-hex + H), 417.3 (M-H_2_O-hex-hex-xyl-hex- hex + H), 163.06 (M-hex-hex-xyl-hex-hex-furostanol + H), which further confirmed that the isolated saponin is uttroside B with a molecular weight of 1215.34 Da (Supplementary Figures S2, S3 and S4).

### Uttroside B exhibits maximum toxicity towards liver cancer cells compared to cancer cells of other anatomical origin

**Figure 3:**
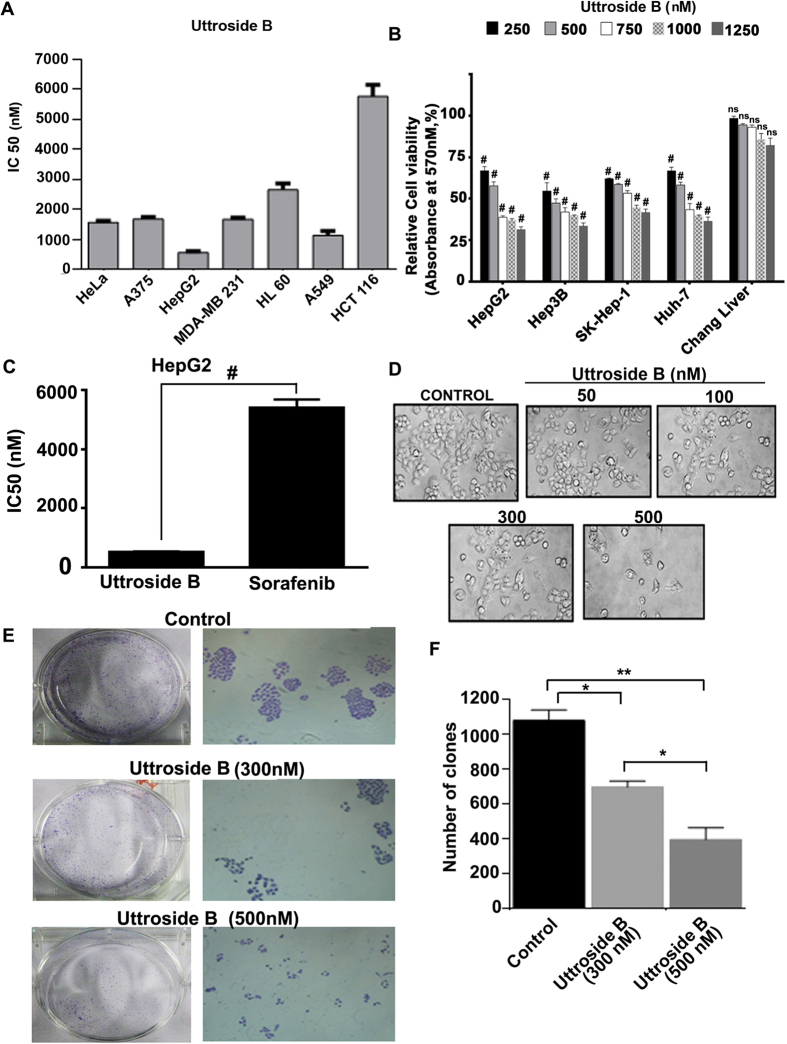
Uttroside B displays maximum sensitivity towards liver cancer cells. (**A**) Comparison of IC50 of uttroside B in a panel of cancer cells of different origin. The cancer cell lines HeLa, A375, HepG2, MDA-MB-231, HL 60, A549 and HCT116 were treated with uttroside B as indicated, incubated for 72 h and the cell viability was estimated by MTT assay. (**B**) Dose dependent effect of uttroside B in the liver cancer cell lines and in the normal immortalized hepatocytes. The liver cancer cell lines HepG2, Hep3B, SKHEP-1, Huh-7 and the normal hepatocytes, Chang liver were treated with uttroside B incubated for 72 h and the cell viability was assessed by MTT assay. The error bars represent ±S.D. Two-way ANOVA followed by Tukey’s post hoc *t* test analysis was used for statistical comparison between control and treatment groups. ^#^P ≤ 0.001, ns non significant. (**C**) Comparison of IC50 of uttroside B with sorafenib. HepG2 cells were treated with uttroside B as indicated, incubated for 72 h and the cell viability was assessed by MTT assay. (**D**) Morphological changes induced by uttroside B in HepG2 cells. HepG2 cells were treated with uttroside B as indicated, incubated for 72 h and photomicrographed (**E**) Uttroside B inhibits the clonogenic potential of HepG2 cells. HepG2 cells were treated with different concentrations of uttroside B for 72 h and clonogenic assay was performed. The error bars represent ±S.D. One-way ANOVA followed by Tukey’s post hoc *t* test analysis was used for statistical comparison between different groups. ^#^P ≤ 0.001. (**F**) Efficacy of uttroside B in inhibiting the clonogenic potential of HepG2 cells. The clones developed were counted and plotted as a graph. Colony containing more than four cells was counted as one clone. The error bars represent ±S.D. One-way ANOVA followed by Tukey’s post hoc *t* test analysis was used for statistical comparison between different groups. **P ≤ 0.01; *P ≤ 0.05.

Uttroside B was screened for its cytotoxicity against a panel of seven human cancer cells of different origin *viz.* skin cancer (A375), liver cancer (HepG2), colon cancer (HCT-116), leukemia (HL60), cervical cancer (HeLa), breast cancer (MDA-MB-231), and lung cancer (A549). The cells were treated with different concentrations of uttroside B (50–10000 nM) for 72 h and the cell viability was evaluated by MTT assay. Interestingly, HepG2 (liver cancer) cells showed maximum sensitivity to this compound with an IC50 of 500 nM followed by A549 (1 μM), HeLa (1.5 μM), A375 (1.6 μM), MDA-MB-231 (1.6 μM), HL60 (2.5 μM), and HCT-116 (6 μM) [Fig. 3(A)]. Further, we evaluated the cytotoxicity of uttroside B in different hepatocellular carcinoma cells (HepG2, Hep3B, SK-Hep1, and Huh-7) and compared with that of normal immortalized hepatocytes (Chang liver) using MTT assay. While different liver cancer cell lines did not exhibit a drastic difference for their sensitivity towards uttroside B (IC50 400–600 nM), 70% of the normal immortalized hepatocytes (Chang Liver) were found viable even at concentrations as high as 1250 nM [Fig. 3(B)]. For further studies, HepG2, the most sensitive among the panel of liver cancer cell lines was selected. The cytotoxicity of uttroside B was compared with that of sorafenib, the only FDA approved drug for liver cancer. Surprisingly, uttroside B was observed almost 12 fold efficacious than sorafenib in inducing cell death [Fig. 3(C)].

HepG2 cells were examined for morphological changes by Phase contrast microscopy, 72 h after treatment with uttroside B. Nuclear condensation, membrane blebbing and formation of apoptotic bodies, which are characteristics of apoptosis was observed in a dose dependent manner in the uttroside B-treated HepG2 cells [Fig. 3(D)]. Further we performed clonogenic assay, which is an *in vitro* assay routinely used as a technique for studying the effectiveness of specific agents on the survival and proliferation. Interestingly, uttroside B induced a drastic dose-dependent reduction in both number and size of the colonies formed, clearly demonstrating its anti-clonogenic potential [Fig. 3E,F].

### Uttroside B induces apoptosis in HepG2 cells, but does not influence any phase of cell cycle

**Figure 4:**
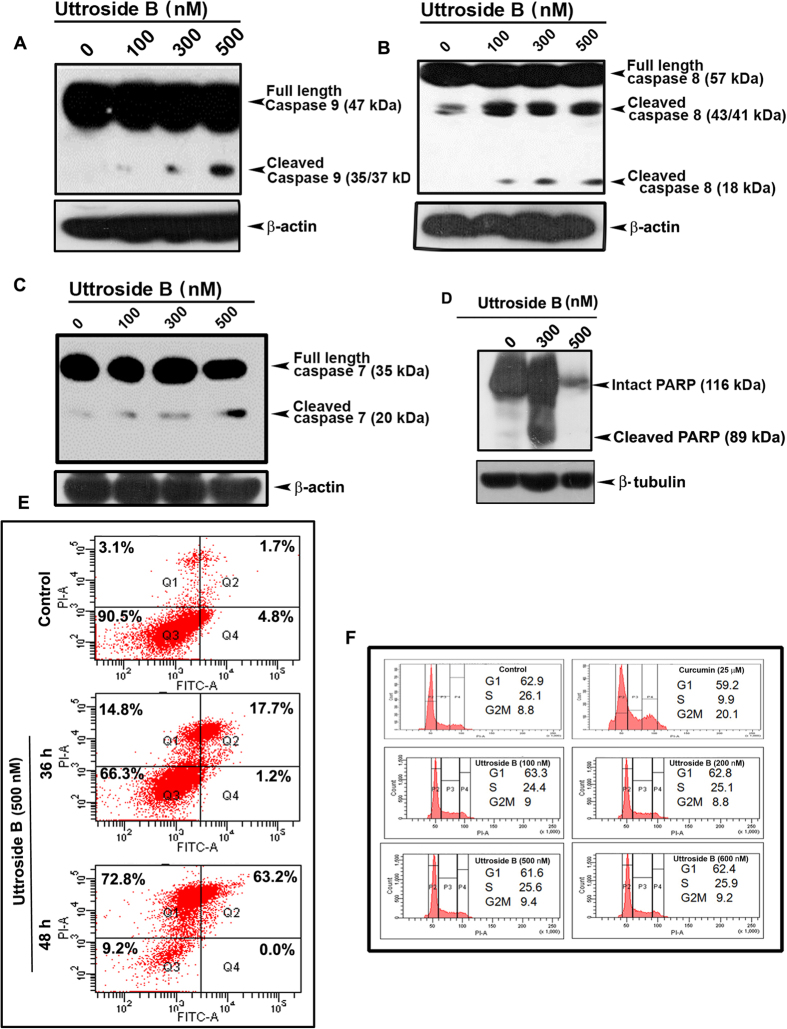
Uttroside B triggers caspase-dependent apoptosis leading to PARP cleavage in HepG2 cells, independent of cell cycle. (**A–F**) Western blots showing caspase activation in HepG2 cells. Whole-cell lysates (WCL) were prepared after treating HepG2 cells with indicated concentrations of uttroside B for 48 h and were resolved on a 15% gel and subjected to Western blotting using antibodies against the caspases 9,8 and 7 detected by ECL. HepG2 cells were treated with uttroside B for 48 h at different concentrations and the WCL were resolved on an 8% gel, immunoblotted against anti-PARP and detected by ECL. (**E**) HepG2 cells were treated with uttroside B for 36 h and 48 h, stained with fluorescein isothiocyanate (FITC)-conjugated Annexin V and propidium iodide and subjected to flow cytometry. The population of Annexin/PI-positive cells in the top right and bottom right quadrants represents the total percentage of apoptotic cells. (**F**) Uttroside B had no effect on any cell cycle phases in HepG2 cells. HepG2 cells were treated with uttroside B for 48 h, stained with propidium iodide and the cell cycle analysis was done using Flow cytometry. Curcumin 25 μM (24 h) was used as positive control.

Uttroside B was found to induce the cleavage of procaspase 9, into its active fragments (p35/37 and p17) in a concentration-dependent manner [Fig. 4(A)]. Similarly, the cleavage of procaspase 8 to its active fragments (p43/41) [Fig. 4(B)], and procaspase 7 to its active fragment (p-20) were also enhanced due to uttroside B treatment after 48 h in a concentration dependent manner [Fig. 4(C)]. As expected, uttroside B induced cleavage of PARP, a downstream event of caspase activation. In uttroside B treated cells, there was a strong cleavage of the mother band to its daughter bands, which were completely degraded at IC50 concentration (500 nM) [Fig. 4(D)]. Further, the efficacy of uttroside B in inducing apoptosis in HepG2 cells was analyzed by Flow cytometry. The results indicate that uttroside B-treated cells stained with fluorescein isothiocyanate conjugated Annexin V and propidium iodide displayed a very significant increase in the percentage of apoptotic cells in a concentration dependent manner compared to control [Fig. 4(E)]. However, the treatment of HepG2 cells with uttroside B did not show any significant effect on cell cycle even after 48 h, at any of the concentrations studied as assessed by Flow cytometry, while the positive control (25 μM) curcumin readily induced cell cycle arrest at G2/M after 24 h [Fig. 4(F)].

### Uttroside B inhibits MAPK and mTOR signaling, but does not affect NF-κB in HepG2 cells

**Figure 5:**
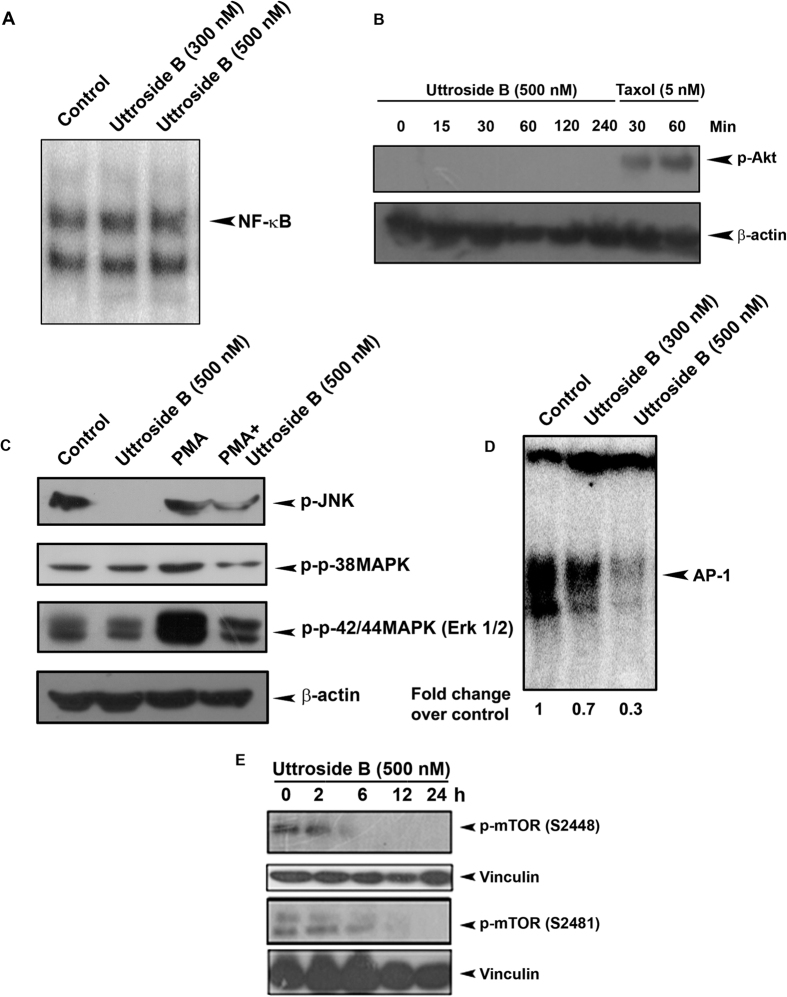
Uttroside B inhibits MAPK and mTOR signaling, the major survival signals in HCC. **(A)** Uttroside B did not cause nuclear translocation of NF-кB. **(B)** Kinetics of uttroside B-induced phosphorylation of Akt in HepG2 cells. HepG2 cells were treated with uttroside B for different time intervals (0–240 min) and the whole cell lysate was resolved on a 10% gel and immunoblotted against phospho-Akt antibody. Taxol was used as a positive control. **(C)** Uttroside B down-regulated the constitutive and PMA-induced phosphorylation of p-JNK, p-p38 & p-p42/44(Erk1/2). **(D)** Uttroside B significantly down-regulated the nuclear translocation of AP-1. **(E)** Kinetics of uttroside B-induced phosphorylation of p-mTOR in HepG2 cells. HepG2 cells were treated with uttroside B at different time intervals and the whole cell lysate was resolved on an 8% gel and immunoblotted against phospho-mTOR (2448) and phospho-mTOR (2481) antibodies.

To study the effect of uttroside B on some of the major signalling events associated with liver cancer progression, the cells were treated with uttroside B and the nuclear extracts and whole cell lysates (WCL) were prepared. Though there was a constitutive activation of NF-κB in HepG2 cells, uttroside B did not produce any significant down-regulation of the same as assessed by the electrophoretic mobility shift assay (EMSA, Fig. 5A). Activation status of Akt was also assessed in these cells by Western Blot. However, no basal activation of Akt was observed in HepG2 and hence, no significant role could be expected for uttroside B (Fig. 5B). Interestingly, the basal activation of MAPK pathway was evident in these cells, among which the activation of p-JNK was significantly down-regulated by uttroside B. Moreover, PMA-induced activation of p-42/44 was also down-regulated by uttroside B suggesting a significant role for this pathway in regulating the anticancer potential of uttroside B against liver cancer [Fig. 5(C)]. Supporting this observation, AP-1, the downstream target of MAPK signalling was also down-regulated by uttroside B as demonstrated by electrophoretic mobility shift assay [Fig. 5(D)]. mTOR pathway is a major survival signal, which plays a pivotal role in cell growth and metabolism and is up-regulated in almost 50% of liver cancer. A strong basal activation of this pathway as assessed by phosphorylation of m-TOR at 2448 and 2481 phosphorylation sites, a read out of p-mTOR activation was observed in HepG2 cells. Interestingly, a time dependent decrease in p-mTOR activation was noticed on uttroside B exposure [Fig. 5(E)].

### Uttroside B is pharmacologically safe as validated by acute and chronic toxicity models

**Figure 6:**
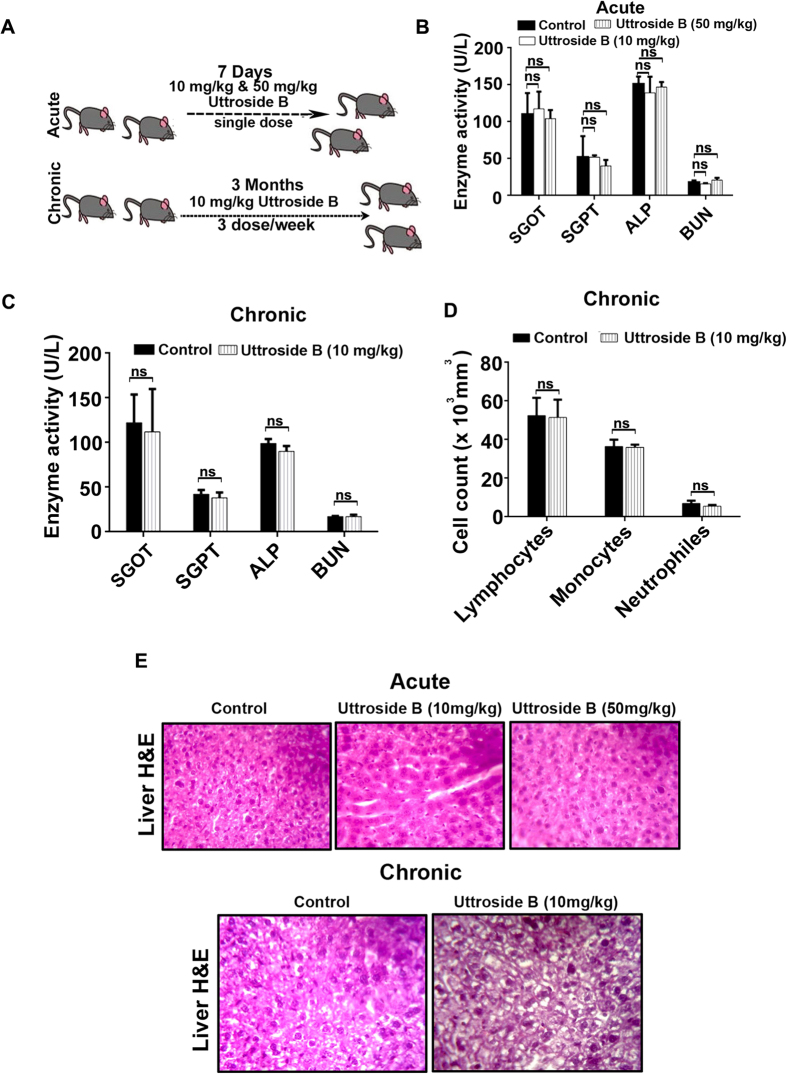
Toxicological evaluation of uttroside B in Swiss Albino mice. **(A)** Schematic representation of toxicity studies using uttroside B in Swiss albino mice. **(B–D)** Serum biochemical analysis of uttroside B illustrates that it does not induce any hepatotoxicity, haematotoxicity, & nephrotoxicity **(E)** Uttroside B does not induce liver toxicity as assessed by liver histopathological analysis. For Histopathological evaluation, tumor tissue isolated from control and uttroside B treated group of Swiss Albino mice was fixed using formalin and cryosectioned before subjecting it to H&E staining. Data represent three independent sets of experiments. The error bars represent ± S.D. Statistical significance was analysed by Student’s t test. ns: non significant.

To rule out the possibility of any toxic side effects due to uttroside B, a detailed toxicological evaluation of the compound was conducted in *Swiss albino* mice as shown in Fig. 6(A). Since the IC50 of uttroside B was 500 nM, the corresponding dose for animal studies was calculated as 10 mg/Kg using the formula, Dose *in vivo* = XY, where X = IC50 (*in vitro*) x molecular weight of the compound and Y = body weight x water content of the animal (0.6 for mice). Another group with 5 times the effective value (50 mg/kg) was also included. The group of mice, which received 10 mg/kg and 50 mg/kg doses of uttroside B did not exhibit any abnormal behaviour and did not show any deviation in serum AST (Aspartate aminotransferase), ALT (Alanine transferase) and ALP (Alkaline phosphatase) [Fig. 6(B)] which are clear markers of abnormality in liver function and in the level of blood urea nitrogen (BUN), which is a marker of nephrotoxicity. In chronic cytotoxic study, the haematotoxicity, hepatotoxicity and nephrotoxcity due to uttroside B was assayed by analyzing the level of total and differential count of WBC and serum levels of AST, ALT, ALP and BUN, respectively in control and treated mice [Fig. 6C,D]. There was no significant difference in any of these parameters from their normal range, indicating that uttroside B is pharmacologically safe and non-toxic. The histopathological analysis of liver tissue isolated from mice administered with uttroside B at the dose same as that used for the tumor reduction studies (10 mg/kg), did not bring in observation of any morphological change characteristic of toxicity. In the liver tissues of acute toxicity study, where a five fold increase in dose compared to that of treatment was used (50 mg/kg), micro vesicular fatty changes were observed, which are reversible changes associated with any chemotherapy. These observations confirm that uttroside B can be safely used as a chemotherapeutic drug for being validated through clinical trials [Fig. 6(E)].

### Uttroside B inhibits development of hepatic xenograft tumor in NOD-SCID mice

**Figure 7:**
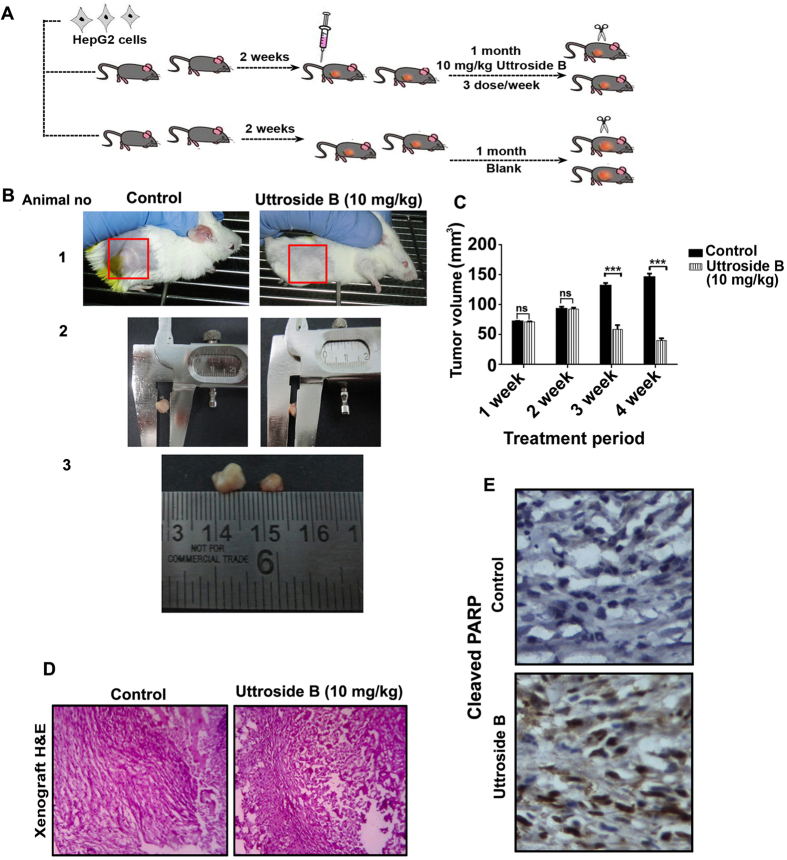
Uttroside B inhibits development of hepatic xenograft tumor in NOD-SCID mice. (**A**) The pictorial representation illustrating the plan of antitumor experiment. **(B)** Representative photographs of mice bearing HepG2 xenograft tumors with or without uttroside B treatment after four weeks**. (C)** Uttroside B effectively inhibited the tumor volume in NOD-SCID mice model. The average volume of HepG2 xenograft tumors among control and uttroside B treated group are shown. Data shows the average of three independent set of experiments with 9 animals per group (P-values < 0.001). **(D)** Histopathological evaluation of tumor tissue isolated from control and uttroside B-treated group of NOD-SCID mice. Formalin fixed cryosections were stained with haemotoxylin and eosin. **(E)** Uttroside B induces apoptosis in liver tumor xenografts IHC analysis of tumor cryosections of control and uttroside B-treated mice using cleaved PARP antibody and the expression of cleaved PARP was detected in tumor tissue sections from mice treated with uttroside B, illustrating apoptosis. Data represent three independent sets of experiments. The error bars represent ± S.D. Statistical significance was analysed by Student’s t test. ns: non significant.

Further, we attempt to validate the anticancer efficacy of uttroside B against hepatic cancer, using an *in vivo* HepG2*-*xenograft model in NOD-SCID mice. The HepG2 cells suspended in matrigel were subcutaneously injected in the flank region of the mice. The experimental plan is schematically represented in Fig. 7(A). Uttroside B, dissolved in PBS, was administered after 15 days of tumor cell implantation when the tumor attained a size of 50–100 mm^3^ approximately. Uttroside B (10 mg/kg bw) was injected intraperitoneally, thrice weekly for four weeks. Group 1 comprised of control animals, which did not receive any treatment. The size of the tumor was measured using Vernier callipers every week and the corresponding tumor volume was calculated. The volume of tumor developed in animals that received uttroside B was significantly low compared to that of the control mice which were injected with the vehicle. At the end of the treatment period, no tumor was visible externally in the group of mice which received uttroside B, whereas in the control group, measurable tumor was developed. However, upon sacrifice, very small tumors were observed beneath the skin of animals treated with uttroside B too, however the size was significantly less compared to that of control animals [Fig. 7B,C]. The tumor mass developed was histopathologically analyzed using H&E staining, which indicated a massive destruction of cells in uttroside B-treated tumor tissue, which correlated with the drastic tumor reduction [Fig. 7(D)]. IHC staining of the formalin fixed cryosections of ectopically implanted human liver xenografts in NOD-SCID mice against cleaved PARP specific antibody revealed the *in vivo* apoptotic effect of uttroside B. Significant up-regulation in the level of cleaved PARP was observed in tumor sections from mice treated with uttroside B [Fig. 7(E)].

## Discussion

Traditional medicines comprising of plant bioactives are often used to treat different types of cancers^11^,^12^,^13^. Steroidal glycosides and triterpenes, commonly known as saponins are a group of compounds abundant in plants and have been reported to be cytotoxic to cancer cells of different origins^14^,^15^,^16^. Degalactotigonin, uttroside A and uttroside B are the cytotoxic saponins isolated from *S. nigrum* Linn^8^,^9^, which is a widely used herb in the Indian traditional systems of medicine. Uttroside B has been isolated from other plants too and has been shown to induce cytotoxicity in neuronal, colon and cervical cancer cells^9^,^10^. Nevertheless, no further information has been reported regarding its mechanism of action or biological safety. In this study, we demonstrate for the very first time, the promising anticancer potential of uttroside B against hepatocellular carcinoma, with mechanism-based evidence (Patent Application No. 201641018401).

It was much compelling to note that the compound triggers cytotoxicity in all liver cancer cells, irrespective of their HBV status, which is often regarded as a common risk factor in regulating hepatocellular carcinoma. In concordance with the currently acceptable dogma regarding apoptosis^17^, uttroside B induced all classical markers of caspase-dependent apoptosis, as evidenced by presence of apoptotic bodies and cleavage of caspases and PARP. However, Parvispinoside B, another saponin, which structurally differs from uttroside B by just one sugar moiety in the lycotetraosyl unit, exhibits strong cytotoxicity against the U937 leukemia cell line^18^, while being not effective against HepG2 cells (IC50 > 100 μM), indicating the significance of the quantity and kind of sugar present in the saponin in determining its anticancer efficacy.

Induction of cell cycle arrest is a vital mechanism through which chemotherapeutic drugs elicit cytotoxicity in cancer cells. However, uttroside B did not exhibit any influence on the cell cycle. According to previous reports, some steroidal saponins such as diosgenin, and smilagenin arrest cell cycle in G0/G1 phase, while some others like tigogenin have no effect on cell cycle indicating that the difference in the spatial conformation of the A- and B-rings and the presence or lack of 5, 6-double bond are not the determinants of the mode of action of the saponin on the cell cycle^2^,^19^.

We also explored the most probable mechanisms behind the anti-cancer potential of uttroside B against liver cancer cells. The tumor survival in HCC involves, highly complex multiple signalling mechanisms that regulate its growth. NF-κB, Akt, MAPK and mTOR are the most prevalent survival signals promoting the progression of hepatic cancer and most of the drugs targeting this cancer are inhibitors of these pathways^20^. Uttroside B could not produce any significant down-regulation in the constitutive activation of NF-κB in HepG2 cells, eliminating the role of NF-κB as a regulator of uttroside B-mediated anticancer effect. Another interesting observation was the absence of activated Akt in HepG2 cells, which prompted us to exclude Akt too from the list. However, basal level phosphorylation of both MAPK and mTOR pathways, which are constitutively activated in HepG2 cells, underscores their regulatory role in these cells. Constitutive activation of JNK, a key regulator of MAPK pathway and constitutive phosphorylation of mTOR pathway was completely abolished by uttroside B, exemplifying the strong role of both these pathways in regulating uttroside B-mediated anticancer potential. Uttroside B also down-regulated PMA-induced activation of p-p42/44(Erk1/2), another important molecule regulating MAPK signalling, reinforcing the role of this pathway in uttroside-B mediated anticancer effect.

Reports indicate that the mammalian target of rapamycin (mTOR) pathway is abnormally activated in a proportion of HCC patients and inhibition of mTOR can suppress liver tumor growth and metastasis^21^,^22^,^23^. Moreover, an up-regulation of mTOR is frequently observed in cholangiocarcinoma, the second most common primary cancer of the liver. A complex interplay between mTOR and MAPK pathways has also been demonstrated during hepatocarcinogenesis^24^. However, further studies are required in order to confirm whether both these pathways are dependent or independent of each other in regulating the anticancer potential of uttroside B.

The biological safety of uttroside B was evaluated in Swiss albino mice using acute and chronic toxicity models. Almost all currently available chemotherapy regimens for the treatment of cancer are associated with considerable systemic toxicities, thus limiting their clinical benefits. The major complication associated with chemotherapy is the reduction in the count of hematological parameters such as lymphocytes, neutrophils and monocytes. Uttroside B produced no significant difference in such hematological parameters confirming that it does not generate any significant toxicity or immunosuppression in animals. Following the onset of liver damage, ALP, AST and ALT are released from the damaged cells, elevating their levels in the serum. Uttroside B did not induce elevation of any of these enzymes in both acute and chronic toxicity studies illustrating its pharmacological safety. Moreover, the normal level of BUN confirmed that uttroside B does not produce any severe toxicological manifestations in the kidney. Additionally, uttroside B, up to 5 fold dose, failed to exhibit any sign of cumulative adverse response in experimental animals as concluded from gross measures such as loss of body weight, ruffling of fur and change in behaviour and food intake underscoring its biological safety. The drastic inhibition of tumor growth produced by uttroside B in NOD-SCID mice bearing human liver cancer xenografts, illustrates the chemotherapeutic efficacy of uttroside B, which was further authenticated by immunohistochemical analysis of the tumor sections for the expression of cleaved PARP, a classical marker of apoptosis.

## Conclusion

Chemotherapeutic options for liver cancer are limited and the prognosis of HCC patients remains dismal. Sorafenib, derived from a *de novo* combinatorial approach by high-throughput screening and approved by US-FDA in 2007, is the only drug currently available for the treatment of hepatocellular carcinoma. It is a multi kinase inhibitor, which can prolong the survival rate up to 20% and the only systemic agent approved for treating advanced, unresectable HCC on the basis of two phase III clinical trials. However, it has been reported to have severe side effects. In the present study, we have isolated uttroside B from the leaves of *Solanum nigrum* Linn and have demonstrated that it is several times more potent than Sorafenib, and does not cause noticeable side-effects *in vitro* and *in vivo*. Drastic inhibition of tumor growth produced by uttroside B in NOD-SCID mice bearing human liver cancer xenografts demonstrates the chemotherapeutic efficacy of uttroside B. These results warrant further clinical evaluation of uttroside B, so that it can be used as an effective chemotherapeutic against liver cancer, which currently has minimal therapeutic options.

## Methods

### Collection and authentication of plant materials

Fresh plants were collected from local areas of Thiruvananthapuram, Kerala and were identified by Dr. G. Valsaladevi, Curator, Dept of Botany, University of Kerala.

### Extraction, Isolation and Identification of active principle

The powdered leaves were extracted using solvents of increasing polarity and extracts were concentrated under Rotavap (SI methods). The most active methanolic extract was subjected to column chromatography to obtain the active principle. The pure active fraction was subjected to spectral analysis for its identification (SI methods).

### Cell lines

All cell lines were procured from NCCS, Pune (SI methods).

### Chemicals and Antibodies

Antibodies against Caspases, β-actin, p-p42/44, p-JNK, p-p38, p-Akt, p-mTOR and Vinculin were obtained from Cell Signaling Technologies (Beverly, MA, USA) and the antibody against PARP was purchased from Santa Cruz Biotechnology (Santa Cruz, CA, USA). All the chemicals used for extraction, column chromatography and high performance liquid chromatography (HPLC) were obtained from Merck Ltd, Mumbai, India. All other chemicals were purchased from Sigma Chemicals (St. Louis, MO, USA) unless otherwise mentioned.

### MTT assay

Antiproliferative activity of the extracts and the isolated compound were evaluated using MTT assay as described earlier^25^ (SI methods).

### Clonogenic assay

The clonogenic cell survival assay was performed as described previously^26^ (SI methods).

### Western blot analysis

The whole cell lysate was prepared from the cells treated with or without drug and subjected to Western blot analysis as described before^27^ (SI methods).

### Flow cytometry

Cell cycle analysis was performed^28^ as described in SI methods.

### EMSA

Nuclear extracts of the cells treated with or without the compound were prepared and EMSA was conducted to evaluate DNA-binding activity of NF-κ B as described earlier^27^,^29^ (SI methods).

### *In vivo* studies

The methods were carried out in accordance with the CPCSEA guidelines and all experimental protocols were approved by Institutional Animal Ethical Committee of Rajiv Gandhi Centre for Biotechnology (IAEC No: 151 (a and b)/RUBY/2012). Toxicological evaluation were conducted in Swiss albino mice as described earlier^28^ (SI methods). HepG2 xenografts models were established in NOD-SCID mice as described in literature^30^. Male NOD-SCID (NOD.CB17-Prkdc^*scid/J*^) mice of age 6–8 weeks were used for the experiment.

### Histopathology and Immunohistochemistry

The mice tissues were collected and cryosectioned. Immunohistochemical analysis of various proteins in the xenograft tumor tissue sections was performed using the detection kit, as per manufacturer’s protocol Super Sensitive Polymer-HRP IHC Detection System (Biogenex, CA, USA) (SI methods).

### Statistical Analysis

The error bars represent ±S.D., taken from independent experiments. Statistical significance was analysed by one way ANOVA or two-way ANOVA followed by Turkey’s post test. Significance level was set at P < 0.05.

## Additional Information

**How to cite this article**: Nath, L. R. *et al*. Evaluation of uttroside B, a saponin from *Solanum nigrum* Linn, as a promising chemotherapeutic agent against hepatocellular carcinoma. *Sci. Rep.* **6**, 36318; doi: 10.1038/srep36318 (2016).

**Publisher’s note:** Springer Nature remains neutral with regard to jurisdictional claims in published maps and institutional affiliations.

## Supplementary Material

Supplementary Information
