# Author Correction: Evaluation of uttroside B, a saponin from *Solanum nigrum* Linn, as a promising chemotherapeutic agent against hepatocellular carcinoma

**DOI:** 10.1038/s41598-020-77440-0

**Published:** 2020-11-18

**Authors:** Lekshmi R. Nath, Jaggaiah N. Gorantla, Arun Kumar T. Thulasidasan, Vinod Vijayakurup, Shabna Shah, Shabna Anwer, Sophia M. Joseph, Jayesh Antony, Kollery Suresh Veena, Sankar Sundaram, Udaya K. Marelli, Ravi S. Lankalapalli, Ruby John Anto

**Affiliations:** 1 Division of Cancer Research, Rajiv Gandhi Centre for Biotechnology, Thiruvananthapuram, Kerala 695014 India; 2 Chemical Sciences and Technology Division, CSIR-National Institute for Interdisciplinary Science and Technology, Thiruvananthapuram, Kerala 695019 India; 3 Department of Pathology, Government Medical College, Thiruvananthapuram, Kerala 695011 India; 4 Division of Organic Chemistry, CSIR-National Chemical Laboratory, Dr. Homi Bhabha Road, Pune, 411008 India

Correction to: *Scientific Reports* 10.1038/srep36318, published online 03 November 2016

This Article contains errors.

In Figure 2A, the NMR spectrum of uttroside A is provided instead of uttroside B. The correct Figure 2A appears below as Figure 1.

Consequently, in the Results section,

“The key information pertaining to steroidal furanose ring include H-21 methyl group at δH 0.99 ppm (3H, d, J = 7 Hz), and hemiketal carbon C-22 at δC 112.5 ppm. Owing to complex pattern of signals arising due to sugars in the region between 3 to 4 ppm, the isolated saponin was peracetylated (Fig. 2A).”

should read:

“The key information pertaining to steroidal furanose ring include H-21 methyl group at δH 1.00 ppm (3H, d, J = 7 Hz), and hemiketal carbon C-22 at δC 110.5 ppm. Owing to complex pattern of signals arising due to sugars in the region between 3 to 4 ppm (Fig. 2A), the isolated saponin was peracetylated.”

“MS-MS analysis in negative mode afforded ions at m/z 1081.5 (M-xyl-H), 919.5 (M-hex-H), 757.4 (M-hex-hex-H), and in positive mode afforded ions at m/z 1235.5 (M-H_2_O + K), 1197.5 (M- H_2_O), 1073.4 (M- H_2_O-hexose + K), 741.4 (M- H_2_O-hex-hex-xyl+H), 579.3 (M- H_2_O-hex-hex-xyl-hex+H), 417.3 (M- H_2_O-hex-hex-xyl-hex-hex+H), 163.06 (M-hex-hex-xyl-hex-hex-furostanol + H), which further confirmed that the isolated saponin is uttroside B with a molecular weight of 1215.34 Da (Supplementary Figures S2, S3 and S4).”

should read:

“MS-MS analysis in negative mode afforded ions at m/z 1081.5 (M-xyl-H), 919.5 (M-xyl-hex-H), 757.4 (M-xyl-hex-hex-H), and in positive mode afforded ions at m/z 1235.5 (M- H_2_O + K), 1197.5 (M- H_2_O +H), 1073.4 (M- H_2_O-hex + K), 741.4 (M- H_2_O-hex-hex-xyl+H), 579.3 (M- H_2_O-hex-hex-xyl-hex+H), 417.3 (M- H_2_O-hex-hex-xyl-hex-hex+H), 163.06 (M-hex-hex-xyl-hex-furostanol), which further confirmed that the isolated saponin is uttroside B with a molecular weight of 1215.34 Da (Supplementary Figures S2, S3 and S4).”

Finally, in Figure 3D, the 100 nM image is a duplication of the 300 nM image. The correct Figure 3D appears below as Figure 2.

**Figure 1:**
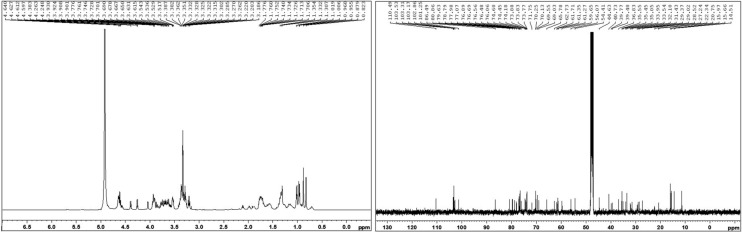
The correct version of Figure 2A.

**Figure 2:**
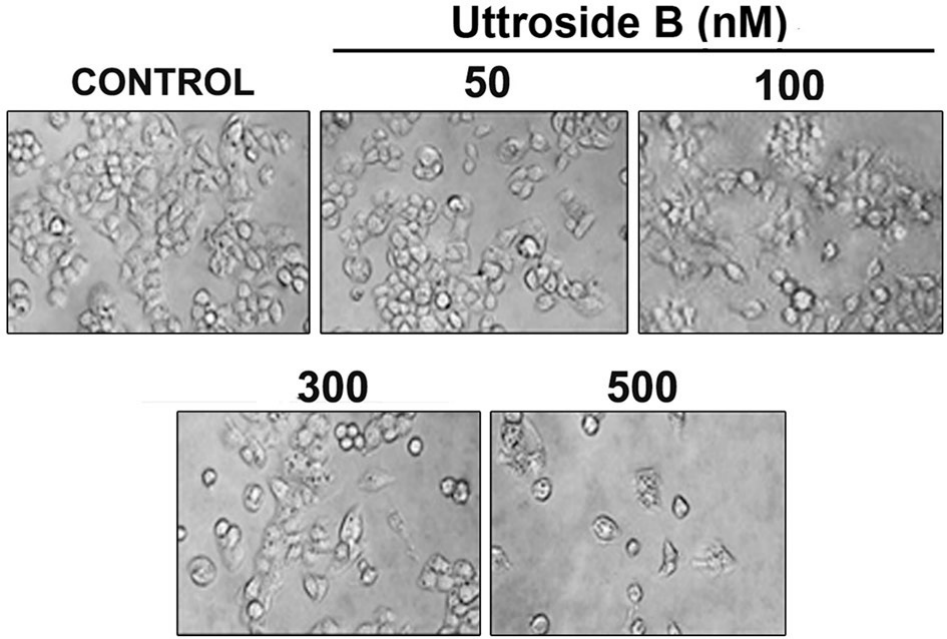
The correct version of Figure 3D.

