# Supplementary material for: Evaluation of uttroside B, a saponin from Solanum nigrum Linn, as a promising chemotherapeutic agent against hepatocellular carcinoma
Source: Sci Rep. 2016 Nov 3;6:36318. doi: 10.1038/srep36318 (PMC5093766; doi:10.1038/srep36318)
Supplement: Supplementary Information [file srep36318-s1.doc]

**SUPPLEMENTARY INFORMATION**

**Evaluation of uttroside B, a saponin from *Solanum nigrum* Linn, as a promising chemotherapeutic agent against hepatocellular carcinoma**

Lekshmi R. Nath,^ɸ,‡^ Jaggaiah N. Gorantla,^ξ,‡^ Arun Kumar T. Thulasidasan,^ɸ^ Vinod Vijayakurup,^ɸ^ Shabna Shah,^ɸ^ Shabna Anwer,^ɸ^ Sophia M. Joseph,^ɸ^ Jayesh Antony,^ɸ^ Kollery Suresh Veena,^ξ^ Sankar Sundaram,^Ψ^ Udaya K. Marelli,^ϑ^ Ravi S. Lankalapalli,*^,ξ^ and Ruby John Anto*^,ɸ^

^ɸ^Division of Cancer Research, Rajiv Gandhi Centre for Biotechnology, Thiruvananthapuram-695014, Kerala, India. E-mail: rjanto@rgcb.res.in; Fax: +91-471-2348096; Tel: +91-471 2529473

^ξ^Chemical Sciences and Technology Division, CSIR-National Institute for Interdisciplinary Science and Technology, Thiruvananthapuram-695019, Kerala, India. E-mail: ravishankar@niist.res.in; Fax: +91-471-2491585; Tel: +91-471-2515352

^Ψ^Department of Pathology, Government Medical College, Thiruvananthapuram-695011, Kerala, India

^ϑ^Division of Organic Chemistry, CSIR-National Chemical Laboratory, Dr. Homi Bhabha Road, Pune-411008, India

* Corresponding authors

‡ These authors contributed equally to this work

**Collection and authentication of plant materials:** Fresh plants were collected in August 2009 from local areas of Thiruvananthapuram, Kerala and were identified by Dr. G. Valsaladevi, Curator, Dept of Botany, University of Kerala, and a voucher specimen has been deposited at Rajiv Gandhi Centre Biotechnology, Division of Cancer Research laboratory (VOUCHER NO: CRP 05).

**General Experimental Procedures:** Silica gel 60 F254 aluminum TLC plates were used to monitor the reactions with short-wavelength ultraviolet light and by charring the TLC plate after spraying with 15% sulfuric acid to visualize the spots. Column chromatography was performed on silica gel 60-120 and 230-400 mesh. Shimadzu HPLC instrument with C18-phenomenex reverse phase column (250 × 21.20 mm, 15µ) was used for purification of semi-purified methanolic extract using gradient grade CH_3_OH and H_2_O. ^1^H and ^13^C NMR spectra were recorded at 500 MHz, 700 MHz and 125 MHz, 176 MHz, respectively. All the spectras were recorded in methanol-d4 (CD_3_OD) and chloroform-d (CDCl_3_). Chemical shifts are given in parts per million and coupling constants in Hz. HR-ESI-MS analysis was performed on a Thermo Scientific Exactive mass spectrometer, with ions given in m/z.

**Cell viability assay using MTT:** MTT [3-(4,5-dimethylthiazole-2-yl)-2,5-diphenyltetrazolium bromide] assay is a standard colorimetric assay suitable for analyzing proliferation, viability, and cytotoxicity. Briefly, HepG2 cells were seeded in 96-well plates (2000 cells/well) and incubated for 24 h. After incubation, cells were treated with different concentrations of organic extracts (25-250 μg/ml) of *S. nigrum* Linn and uttroside B (250-1250 nM) for 72 h. Following the treatment, fresh media containing MTT solution was added to each well (final concentration of 1 mg/mL in PBS) and incubated for 2h. Lysis buffer (20% sodium dodecyl sulphate in 50% dimethylformamide) was added to the wells (0.1 mL/well) followed by 1 h incubation at 37 °C and the optical density was measured at 570 nm using ELISA plate reader (Bio-Rad). The relative cell viability in percentage was calculated as (A_570_ of treated samples/A_570_ of untreated samples) x 100. The IC50 values were extrapolated from polynomial regression analysis of experimental data.^29^

**Clonogenic assay:** The clonogenic cell survival assay is used to determine the long-term fate of proliferating cells. Briefly, 500-1000 cells/well was seeded in 6 well plates and was treated with different concentrations of isolated compounds (uttroside B) for 72 h. After the treatment period media was aspirated, fresh media was added and incubated for 1 week. The clones developed were fixed with glutaraldehyde, stained using crystal violet, counted and graphically plotted.^14^

**Western blot analysis:** Approximately 0.5 x10^6^ cells were grown on 60 mm culture plates and exposed to uttroside B for the indicated time period. After treatment, the cells were lysed in cell lysis buffer [(20 mMTris of pH 7.4, 250 mM NaCl, 2 mM EDTA, 0.1% Triton, 1 mM DTT (1,4-dithiothreitol)] supplemented with protease and phosphatase inhibitors [PMSF (0.5 mM), sodium orthovanadate (4 mM), aprotinin (5 mg/mL) and leupeptin (5 mg/mL)]. The lysates were separated by 10 or 15 % SDS-PAGE and transferred to a PVDF membrane (Hybond-P, GE Healthcare Life science). After the transfer, the membrane was exposed to 5% fat free milk in TBST buffer for 1 h at room temperature to block the nonspecific binding of antibodies, followed by overnight incubation with the primary antibody [1:1000 dilution] in 3% BSA in TBST buffer at 4 °C. Excess antibody was washed off with TBST buffer and incubated with corresponding secondary antibody [1:5000 dilution] coupled with horse radish peroxidase (HRP) in 5% fat free milk in TBST buffer. The bands were visualized using enhanced chemiluminescence kit (Millipore, St Charles, MO, United States) following manufacturer’s protocol.^30^

**Estimation of apoptosis by Annexin V-PI- FACS.** The extent of apoptosis induced by uttroside B was estimated by FACS using an Annexin V apoptosis kit (Santa Cruz, CA, USA). Briefly, cells were seeded in 60 mm culture plates, and incubated with uttroside B for 36h and 48h, cells were trypsinized and pelleted down by low speed centrifugation, washed with PBS and were suspended in 1X assay buffer. To a 400ml mixture, 10 mL of FITC conjugated Annexin V and 5 mL of propidium iodide were added and incubated for 15 min in the dark at room temperature. The cells were then analyzed immediately by flow cytometry to get the % of apoptotic cells (FACS Aria™, BD Bioscience) ^30^

**Flourescence Activated Cell Sorter analysis (FACS):** Cell cycle analysis using flow cytometry was performed to investigate the cell cycle arrest induced by uttroside B. Briefly, 0.5 x10^6^ cells were seeded in 60 mm plates and subjected to uttroside B treatment for 48 h followed by trypsinization and pelleting down. Curcumin (25 μM, 24 h) was used as the positive control. The cell pellets were fixed in 70% ice-cold ethanol, treated with 5 μL (10 mg/mL) RNase A and incubated for 30 min at 37 °C, 10 μL (10 mg/mL) propidium iodide was added, filtered via filter tubes and analyzed using the FACS Aria flow cytometer (BD Biosciences).

**Electrophoretic mobility shift assay (EMSA):**

HepG2 Cells were treated with uttroside B for two hours, scraped and suspended in 150 μL of lysis buffer [HEPES (10 mM), KCl (10 mM), EDTA (0.1 mM), EGTA (0.1 mM), DTT (1 mM), phenylmethylsulfonyl fluoride (0.5 mM), leupeptin (2 μg/mL), aprotinin (2 μg/mL), benzamidine (0.5 mg/mL)] for 30 min, after which 4.5 μL of 10% Nonidet P-40 was added. The pellet was suspended in 25 μL of nuclear extraction buffer [HEPES (20 mM, pH 7.9), NaCl (0.4 M), EDTA (1 mM), EGTA (1 mM), DTT (1 mM), phenylmethylsulfonyl fluoride (1 mM), leupeptin (2 μg/mL), aprotinin (2 μg/mL), benzamidine (0.5 mg/mL)] and centrifuged after 2 h. The nuclear extract collected (8 μg of protein) was used to perform EMSA by incubating it with 16 fmol of 32P end labeled 45-mer double stranded NF-κB oligonucleotide from the human immunodeficiency virus-1 long terminal repeat (5 -TTGTTACAAGGGACTTTCCGCTGGGGACTTTCCAGGGAGGCGTGG-3) and with 1 μg/mL poly(dIdC) in a binding medium for 30 min at 37 °C. The DNA-protein complex was resolved using a 6.6% native polyacrylamide gel and the radioactive bands were visualized by phosphorimaging (Bio-Rad Personal FX)^29,31^.

**Toxicological evaluation:**

**a. Animals:** Six to eight-week-old female *Swiss albino* mice (18-22 g) were obtained from the Animal Research Facility of Rajiv Gandhi Centre for Biotechnology. The methods were carried out in accordance with the guidelines approved by Institutional Animal Ethical Committee of Rajiv Gandhi Centre for Biotechnology (IAEC No: 151 (a)/RUBY/2012)

**b. Acute toxicity study:** *Swiss albino* mice were randomly divided into 3 groups of 6 animals each and were allowed to acclimatize for a week. Group I was taken as the control, which received only vehicle, while Group II and III received a single dose of uttroside B dissolved in PBS (10 mg/kg and 50 mg/kg body weight, respectively). The mice were observed continuously for 1 h, for any gross behavioral changes and death, and then intermittently for the next 6 h and 24 h. The animals were observed frequently for the next 7 days from the day of treatment after which the animals were euthanized in a CO_2_ chamber. The blood serum was collected for analyzing biochemical parameters of liver function, the abnormal values of which are indicative of toxicity. The liver was fixed in 10% buffered formalin and the thin cryostat sections (LEICA CM 1850UV Cryostat) were stained with haematoxylin and eosin for histopathological evaluation.^30^

**c. Chronic toxicity study:** *Swiss albino* mice were randomly divided into 2 groups of 6 animals each and were allowed to acclimatize for a week. Group I received vehicle and Group II received 10 mg/kg body weight of uttroside B. The compounds were given as intraperitonial injection on alternate days, thrice in a week, for 3 months. The animals were observed frequently during this period after which the animals were euthanized in a CO_2_ chamber. The blood serum was collected for analyzing biochemical parameters of liver function, the abnormal values of which are indicative of hepatotoxicity. The liver was fixed in 10% buffered formalin and the thin cryostat sections were stained with haematoxylin and eosin for histopathological evaluation^30^.

**Evaluation of the anticancer activity of uttroside B, isolated from *S. nigrum* Linn in human liver cancer xenograft model:** HepG2 xenografts models were established in NOD-SCID mice as described in literature.^10^ Male NOD-SCID (NOD.CB17-Prkdc*^scid/J^*) mice of age 6-8 weeks were used for the experiment. Tumors were induced by subcutaneous injection of HepG2 cells (7x10^6^ cells in 100 μL matrigel) in the lower right or left flank of mice and were allowed to grow for a period of two weeks to attain a size of approximately 50-100mm^3^ as measured by Vernier calipers. The mice were then randomly grouped into control and treatment group of 9 animals each. The treatment groups were injected intraperitoneally with uttroside B (10 mg/kg doses thrice weekly) for one month. Tumor volume was measured every seven days to evaluate tumor growth and drug response. The animals were sacrificed at the end of the experiment and tumor samples were collected for histopathological and immunohistochemical analysis.^32,33^

**Extraction of plant material:** The leaves of *Solanum nigrum* Linn were dried at room temperature and grounded to coarse powder resulting in 100 g of the material. The powdered material is subjected to maceration in a shaker incubator at 150 rpm using the gradient solvent system: hexane (500 mL), dichloromethane (500 mL), ethyl acetate (500 mL), and methanol (500 mL), which after filtration and concentration yielded 1.7 g, 2.5 g, 4.2 g, and 6.3 g, respectively. Methanolic extract was found to be most active against liver cancer cell lines (Figure S1).


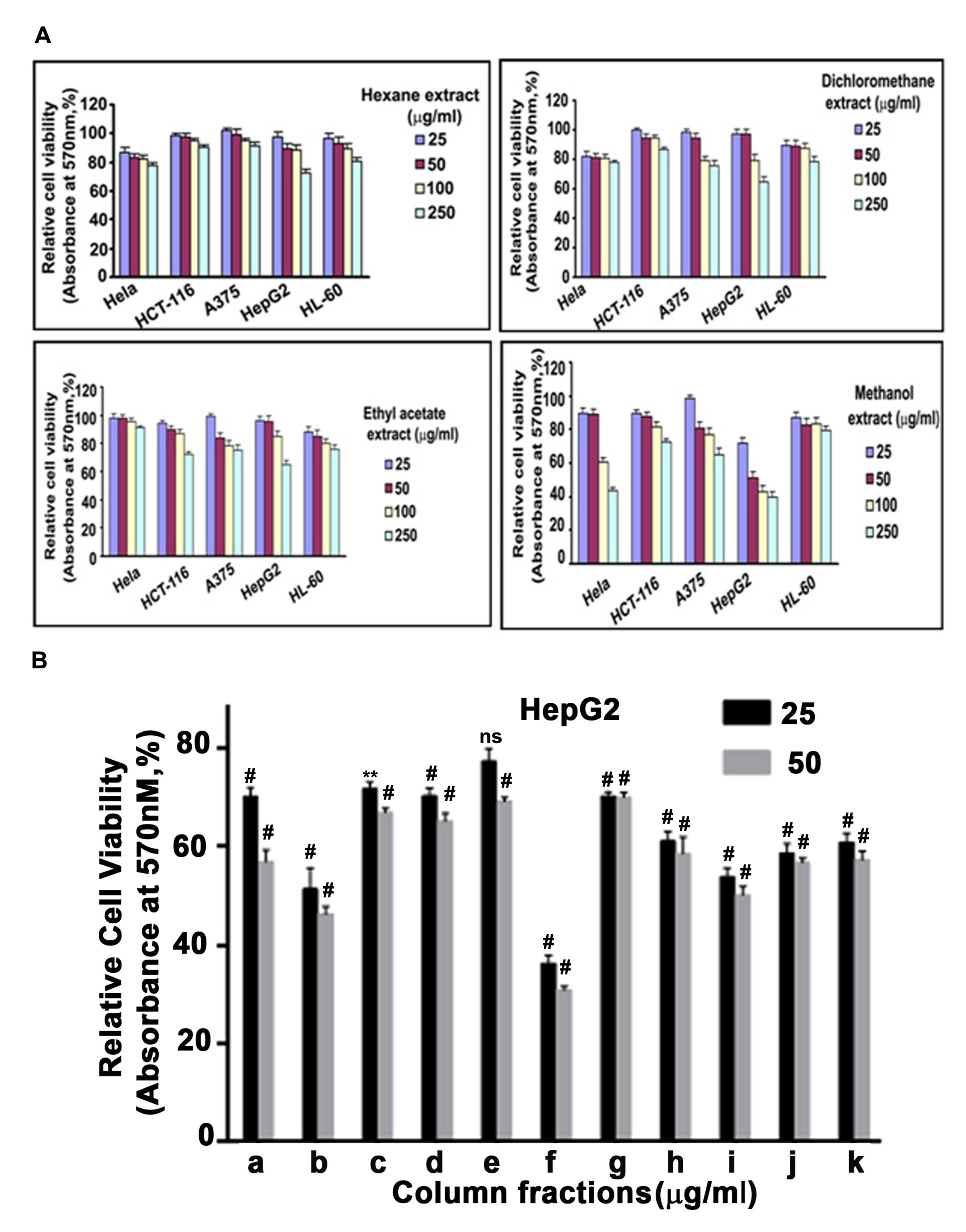


**Figure S1.** **(A)** Cytotoxicity of organic extracts of *S. nigrum* in a panel of five cancer cell lines. The cancer cells were treated with indicated concentrations of hexane extract, dichloromethane extract, ethyl acetate extract and methanol extract, incubated for 72h as indicated and the cell viability was assessed by MTT assay. **(B)** Cytotoxicity induced by *S. nigrum* Linn isolated column fractions, in HepG2 cells. HepG2 cells were treated with different concentrations of column fractions as indicated and cell viability was assessed by MTT. Data represent three independent sets of experiments. The error bars represent ± S.D.

**Isolation and purification of uttroside B:** The methanolic extract (6.3 g) was subjected to fractionation by column chromatography. The column was packed (silica gel 60-120 mesh, 45 cm × 3 cm) with hexane, loaded the compound and eluted using gradient solvent system: hexane/chloroform (500 mL each of 100/0, 80/20, 60/40, 50/50, 40/60, 20/80, 0/100), to chloroform/methanol (500 mL each of 100/0, 95/5, 90/10, 85/15, 80/20, 75/25, 70/30, 60/40, 50/50). Concentration of the fraction eluted during chloroform/methanol (60/40) elution afforded a major polar fraction (1.125 g) which was found to be most active fraction. The polar active fraction (1.125 g) was further subjected to purification by flash column chromatography (silica gel 230-400 mesh, 30 cm × 2 cm). The column was packed with chloroform, loaded the compound and eluted using a gradient solvent system: chloroform (100 mL) to chloroform/methanol (300 mL each of 90/10, 80/20, 70/30). Fraction obtained during elution at chloroform/methanol (70/30) was found to be a mixture of proline and a saponin as observed in ^1^H-NMR (Fig 1A) and appeared to be a pale yellow foamy solid (700 mg) (Fig 1B). The mixture of proline and saponin [SP (700 mg)] was redissolved in H_2_O (6 mL) and then subjected to purification by reverse-phase preparative HPLC, using the following gradient program: solvent A (H_2_O) and solvent B (MeOH), linear gradient 0 min 0% B, 5 min 10% B, 10 min 20% B, 15 min 30% B (isolated proline, 130 mg, between 10-15 min), 20 min 50% B, 30 min 60 % B, 60 min 80% B, 65 min 90% B, 70 min 100% B. The saponin eluted between 65% to 80% B which was monitored by collecting the eluted fractions on a TLC plate and charring with 15% sulfuric acid in ethanol. Concentration followed by lyophilization afforded a white solid uttroside B (120 mg).


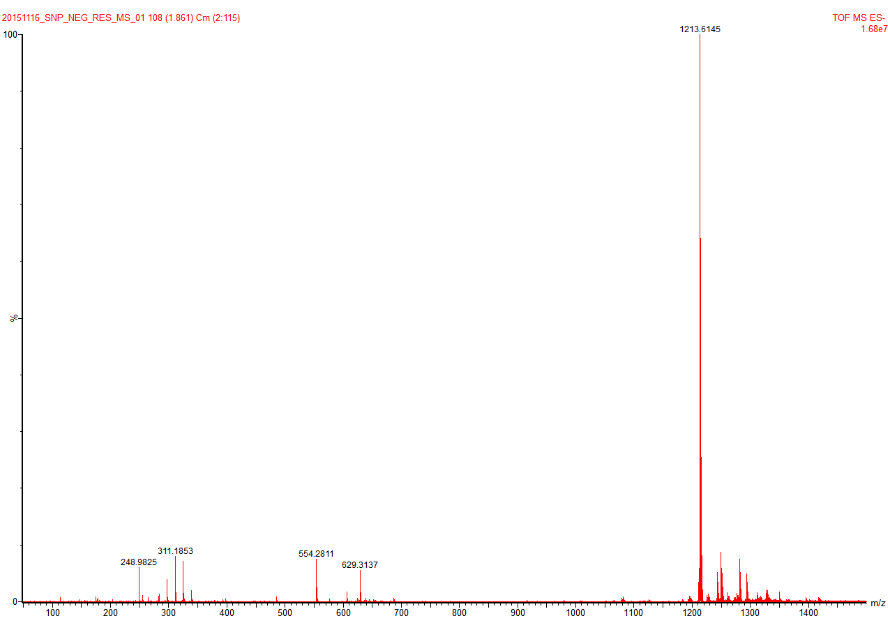


Figure S2 : HRESIMS of uttroside B


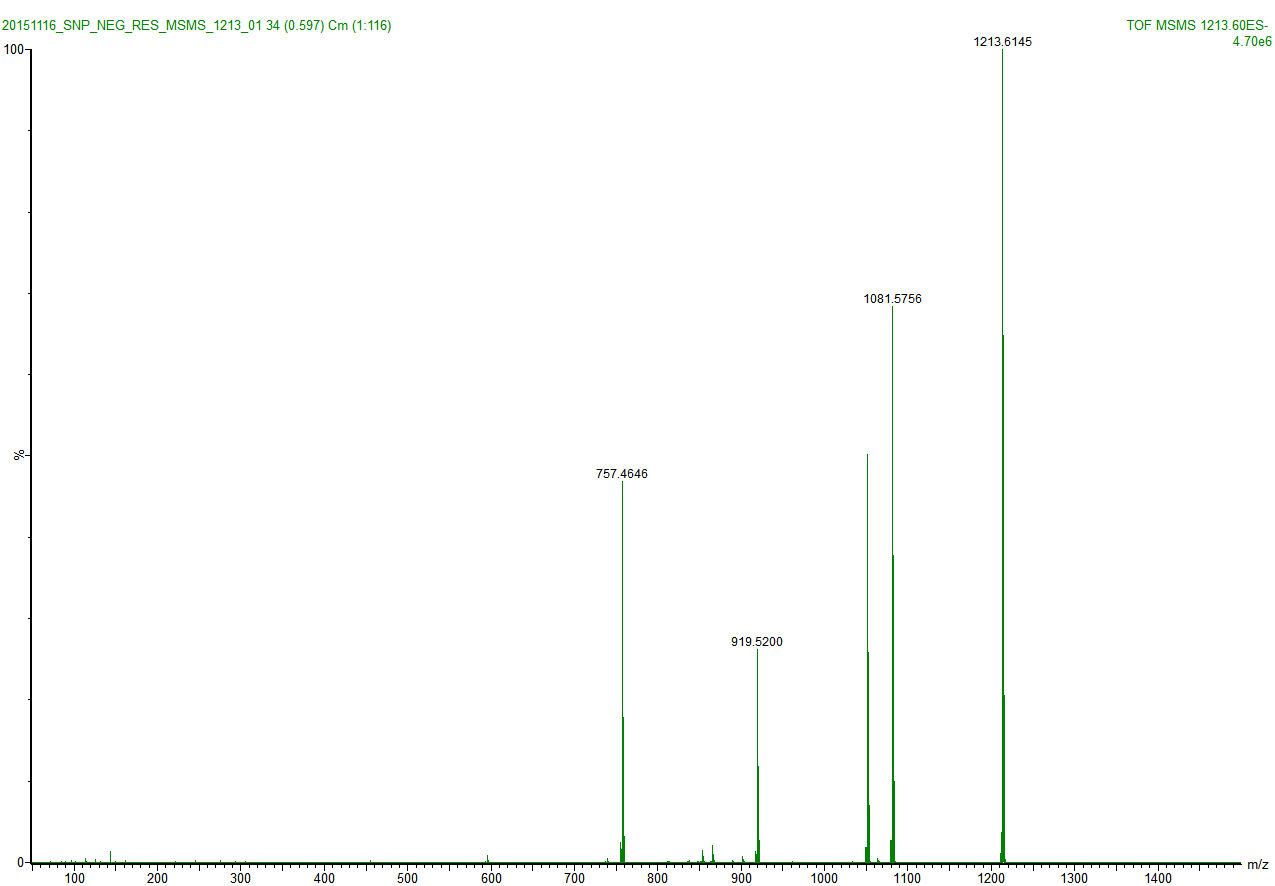


Figure S3 : Negative mode MS-MS analysis of uttroside B


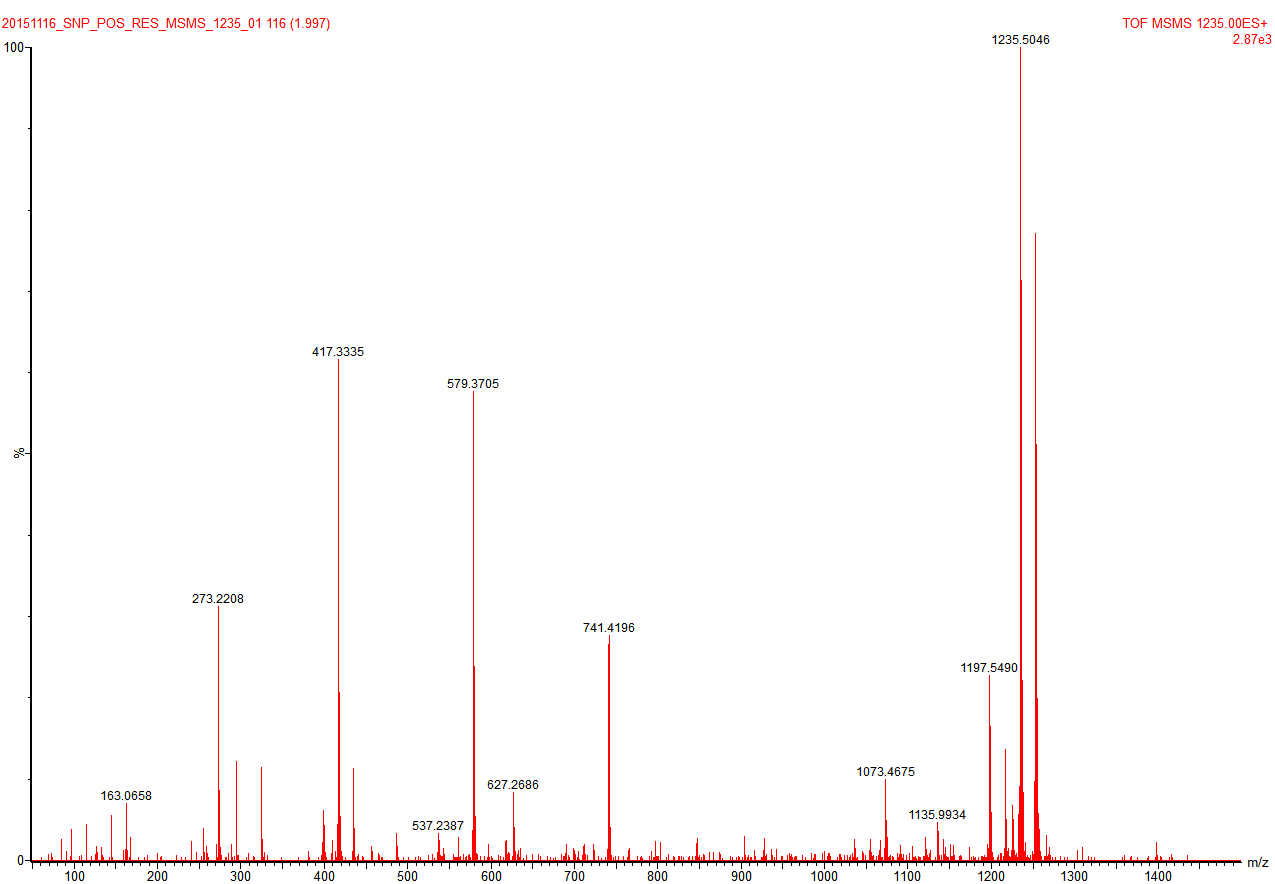


Figure S4 : Positive mode MS-MS analysis of uttroside B

**Peracetylation of saponin**: Isolated saponin (20 mg) was dissolved in 3 mL of pyridine: Ac_2_O (2:1), and then stirred at room temperature under N_2_ atmosphere. After 24 h, the reaction mixture was quenched with aqueous saturated NaHCO_3_ (25 mL), extracted with ethyl acetate (25 mL × 2), dried under Na_2_SO_4_ and concentrated. Purification by column chromatography using hexane/ethyl acetate 60/40 to 40/60 afforded the peracetylated product of saponin (10 mg) as a white solid which was characterized by NMR in CDCl_3_ (Figure 1C, Table S1). HR-ESI-MS [M+Na]^+^ C_88_H_124_O_43_Na of peracetylated product calcd for m/z 1891.7414, found 1891.7382.

Table S1: ^1^H NMR (700 MHz in CDCl_3_), ^13^C NMR (176 MHz in CDCl_3_) data of peracetylated product from saponin

Position δ ^13^C δ ^1^H mult (*J* (Hz)) Position δ ^13^C δ ^1^H mult (*J* (Hz))

1 37.0 0.92 (m) Gal

1.70 (m) 1 99.6 4.45 d (8.4)

2 28.7 1.43-1.45 (m), 1.80 (m) 2 69.6 5.38 app t (9.1)

3 79.2 3.50-3.52 (m) 3 73.4 4.92 dd (2.8, 9.1)

4 34.2 1.21-1.23 (m) 4 72.5 4.05-4.06 (m)

1.51 (m) 5 71.6 3.67-3.68 (m)

5 44.7 1.03 (m) 6 63.4 4.14 (m), 4.23 dd (5.6, 11.9)

6 29.7 1.27 (m) Glc I

7 32.4 0.87 (m), 1.68 (m) 1*ꞌ* 100.92 4.34 d (7.0)

8 34.9 1.49-1.51 (m) 2*ꞌ* 79.1 3.87 app t (8.4)

9 54.3 0.61-0.63 (m) 3*ꞌ* 76.4 3.95 app t (9.1)

10 35.7 4*ꞌ* 68.7 4.99 (m)

11 28.8 1.30-1.33 (m) 5*ꞌ* 71.5 3.46-3.48 (m)

12 39.7 1.17-1.19 (m) 6*ꞌ* 62.2 4.07-4.09 (m)

1.75-1.77 (m) Glc II

13 43.5 1*ꞌꞌ* 99.3 4.85 d (8.4)

14 54.8 0.96-0.98 (m) 2*ꞌꞌ* 72.1 5.20 app t (8.4)

15 34.0 1.36-1.41 (m) 3*ꞌꞌ* 73.2 5.31 app t (9.1)

2.14-2.17 (m) 4*ꞌꞌ* 69.8 5.33 app t (9.1)

16 84.3 4.69-4.72 (m) 5*ꞌꞌ* 72.4 3.75-3.77 (m)

17 64.3 2.45 d (9.8) 6*ꞌꞌ* 63.3 4.27 (m), 4.39 dd (7.7, 11.9)

18 14.1 0.65 (s) Xyl

19 12.2 0.82 (s) 1 97.1 5.16 (m)

20 103.7 2 68.2 4.88 (m)

21 11.6 1.57 (s) 3 67.9 5.02-5.04 (m)

22 151.7 4 67.1 4.77-4.78 (m)

23 23.2 2.08 (m) 5 58.8 3.55 dd (4.2, 12.6), 4.12 (m)

24 30.8 1.25 (m) Glc III

1.52 (m) 1*ꞌꞌꞌ* 100.99 4.48 d (8.4)

25 32.7 1.73-1.75 (m) 2*ꞌꞌꞌ* 71.3 5.01 (m)

26 75.1 3.31 dd (6.3, 9.1) 3*ꞌꞌꞌ* 72.9 5.22 app t (9.1)

3.71-3.72 (m) 4*ꞌꞌꞌ* 68.5 5.11 app t (9.1)

27 16.5 0.90 d (6.3) 5*ꞌꞌꞌ* 71.7 3.70-3.71 (m)

6*ꞌꞌꞌ* 62.0 4.15 (m), 4.28 (m)
